# Value of Transfusion of Blood in Treatment of the Wounded in War

**Published:** 1919-01

**Authors:** 


***The Value of the Transfusion of Blood in the Treatment of the Wounded in War.*** By ALEXANDER PRIMROSE. *Annals of Surgery*, August, 1918.

The author emphasizes the good effects of the transfusion of whole blood. He considers it as a most effective life-saving measure in cases of hemorrhage, both primary and secondary.

He had the opportunity of resorting to it in 10 cases while in Salonika. 9 of the patients made good recoveries, but in one case hemolysis occurred and proved fatal. This of course proves the necessity of preliminary tests of the blood of donor and recipient, to prevent such an occurrence, by determining, if possible, whether or not hemolysis is likely to occur.

Nearly all surgeons now have experimented with the transfusion of blood, and controlled its good results. In the early months of the war, the Canadians established its value in cases of shock accompanied by hemorrhage. Major L. Bruce Robertson, who was on duty in a Canadian Casualty Clearing Station, reports a series of 68 cases in wich transfusion of the blood was resorted to. These cases were classified as follows :

| | |
|---|---|
| *Primary Hemorrhage* : | |
| Life saving. . . . . . . . . . . . . . . . . . . . . | 36 |
| Immediately beneficial but died subsequently . . . | 15 |
| No benefit. . . . . . . . . . . . . . . . . . . . . | 4 |
| Harmful (hemolysis) . . . . . . . . . . . . . . . | 2 |
| *Secondary Hemorrhage* . . . . . . . . . . . . . . | 9 |
| Of these 6 recovered and 3 died. . . . . . . . . . | |
| *Severe Carbon Monoxide Poisoning* . . . . . . . . | 2 |

In these latter cases of carbon monoxide poisoning, venesection with the removal 1 000 cc. of blood in two stages gave but moder-rate relief. On this account, 1 000 cc. of blood was transfused, subsequent improvement was evident, and the patients recovered.

The author agrees with the conclusions of Archibald and Mac Lean that transfusion is useless in cases of shock; but that it proves of immense value where shock is accompanied by hemorrhage.

Whenever the wounded man has lost great quantities of blood, the replacement of that blood is a most effective treatment. After transfusion the patients are in a vastly better position to combat shock, sepsis, the ordeal of all necessary operations, or whatever conditions may threaten life.

The citrate method has many advantages; the use of Kimpton's tubes has been found convenient. Actual methods allow of the keeping of blood drawn from the donor, for 48 hours or more, in cold storage, in expectation of some emergency. Successful results from the transfusion of immunized blood in septic cases (staphylococcus septicemia for instance) have been reported by some authors. This method consists of immunizing the donors by doses of vaccines prepared from the particular virus previous to transfusion.

The quantities of blood transfused range between 600 and 700 cc. in cases of slight hemorrhages, and 1 200 cc. in severe cases. Donors have been found to suffer nothing but very temporary inconvenience.

Two main dangers are to be avoided in the transfusion of blood: namely, the transfusion of some infective disease, and hemolysis.

The donor should be carefully examined for the purpose of excluding syphilis and other infectious diseases.

The blood of both donor and recipient should be tested as to their iso-agglutinin characters. Of course the most severe and possibly fatal results would occur if the serum of the recipient proved to agglutinate the corpuscles of the donor.

The question of prevention of hemolysis by suitable tests has recently received a great deal of attention.

Two iso-agglutinins and two iso-hemolysins may be demonstrated in human blood. Absence of both iso-agglutinins, or the presence of one or both of them in an individual sample of blood, permits the classification of human blood into four main groups. Moss prepared two standard human sera, each serum containing one of the iso-agglutinins. These sera are used for testing the blood of both the donor and recipient. If both of these present similar reactions a suitable donor has been secured. These standard sera may be kept for quite a long time, thus rendering the method a most practical one.

A paper on that subject has been recently published by Captain Karsner. He tested the blood of 1 000 soldiers and classified the results according to Moss classification. He found that in 46.2 per cent. of the cases the corpuscles cannot be agglutinated by any serum. It has been suggested that, in cases of emergency, when tests cannot be made, donors of this group only should be used, so as to avoid that form of hemolysis where the corpuscles of the donor are hemolyzed by the serum of the recipient. In fact, on the front, in advanced posts especially, transfusion may be resorted to even if it is impossible to test for agglutination. The danger of hemolysis, although real, is not so great as to make it unjustifiable to proceed to it in urgent cases, without investigating the characteristics of the blood.

